# Social, structural, behavioral and clinical factors influencing retention in Pre-Exposure Prophylaxis (PrEP) care in Mississippi

**DOI:** 10.1371/journal.pone.0172354

**Published:** 2017-02-21

**Authors:** Trisha Arnold, Lauren Brinkley-Rubinstein, Philip A. Chan, Amaya Perez-Brumer, Estefany S. Bologna, Laura Beauchamps, Kendra Johnson, Leandro Mena, Amy Nunn

**Affiliations:** 1 Department of Medicine, University of Mississippi Medical Center, Jackson, Mississippi, United States of America; 2 Jackson State University, Jackson, Mississippi, United States of America; 3 Department of Social Medicine, University of North Carolina, Chapel Hill, North Carolina, United States of America; 4 Center for Health Equity Research, University of North Carolina, Chapel Hill, North Carolina, United States of America; 5 Warren Alpert Medical School of Brown University, Providence, Rhode Island, United States of America; 6 The Miriam Hospital, Providence, Rhode Island, United States of America; 7 Department of Sociomedical Sciences, Columbia University, New York City, New York, United States of America; 8 Mississippi State Department of Health, Jackson, Mississippi, United States of America; 9 School of Public Health, Brown University, Providence, Rhode Island, United States of America; Agencia de Salut Publica de Barcelona, SPAIN

## Abstract

Pre-exposure prophylaxis (PrEP) is a biomedical intervention that can reduce rates of HIV transmission when taken once daily by HIV-negative individuals. Little is understood about PrEP uptake and retention in care among the populations most heavily impacted by the HIV epidemic, particularly among young men who have sex with men (YMSM) in the Deep South. Therefore, this study explored the structural, social, behavioral, and clinical factors that affect PrEP use and retention in care among YMSM in Jackson, Mississippi. Thirty MSM who were prescribed PrEP at an outpatient primary care clinic were interviewed and included 23 men who had been retained in PrEP care and seven who had not been retained. The mean age of participants was 26.6 years. Most (23) participants were African American. Major factors affecting PrEP use and retention in PrEP care included 1) structural factors such as cost and access to financial assistance for medications and clinical services; 2) social factors such as stigma and relationship status; 3) behavioral factors including sexual risk behaviors; and 4) clinical factors such as perceived and actual side effects. Many participants also discussed the positive spillover effects of PrEP use and reported that PrEP had a positive impact on their health. Four of the seven individuals who had not been retained re-enrolled in PrEP care after completing their interviews, suggesting that case management and ongoing outreach can enhance retention in PrEP care. Interventions to enhance retention in PrEP care among MSM in the Deep South will be most effective if they address the complex structural, social, clinical, and behavioral factors that influence PrEP uptake and retention in PrEP care.

## Introduction

An estimated 1.1 million individuals are living with HIV/AIDS in the United States (US) [1]. Men who have sex with men (MSM) bear a disproportionate burden of the HIV epidemic [2]. Approximately 65% of new HIV infections are attributed to male-to-male sexual contact [3]. HIV incidence continues to increase among MSM, and among Black/African American (AA) young MSM (YMSM) in particular [2, 4, 5]. AA MSM represent less than 1% of the population but account for 25% of new HIV diagnoses (5). AA MSM are three times more likely to be HIV-infected than white counterparts [5].

In 2013, the Southern US accounted for 44% of new HIV diagnoses nationally [6]. Mississippi (MS) ranks 9^th^ highest in rate of new HIV infections [7], and its capital, Jackson, ranks 4^th^ in overall prevalence of HIV [8]. Additionally, Jackson has the highest rate of HIV infections among MSM [9] and, accordingly, the majority of new infections reported in Jackson are among AA YMSM [10]. Given this disproportionate burden of HIV, innovative HIV prevention interventions are particularly important to lower the risk of HIV for AA YMSM in the Deep South, and in Mississippi in particular.

Current HIV prevention approaches have had only modest effects in reducing HIV transmission, particularly among AA MSM. Pre-exposure prophylaxis (PrEP) is a biomedical approach used to prevent HIV transmission. PrEP can dramatically reduce HIV acquisition, particularly when patients take medications regularly [11–14]. The implementation of PrEP has been delivered successfully in diverse clinical settings [15] and its efficacy among MSM, including AA MSM, has been demonstrated in randomized controlled trials and open-label studies [16–21]. Recent preliminary analyses from 32 industry sponsored demonstration projects demonstrate PrEP’s efficacy in real world settings [22]. Until recently, PrEP uptake has been somewhat limited [23–28], and recent analyses highlight disparities in PrEP uptake [29] as AA MSM account for a declining number of all PrEP users.

In addition, PrEP is most effective when patients adhere to medications, and adherence requires retention in PrEP care. PrEP adherence has been studied in the context of clinical trials [11, 30], but little is known about factors associated with adherence or how to enhance retention in PrEP care in real world settings, particularly for AA YMSM. Barriers to PrEP initiation, adherence and retention in care are complex: structural, social, clinical and behavioral factors may affect PrEP use [27, 31]. Structural factors shape HIV risk via institutions, the environment, access to services and policy. Social factors affect HIV risk through interactions with others [24, 25, 32, 33]. Individual factors include those that are behavioral or clinical and are usually related to a person’s decision-making, attitudes, or perspectives. Given the need to better understand the factors that may play a role in PrEP uptake and retention in care, particularly among individuals at disproportionate risk of HIV acquisition, this study investigated social, structural, behavioral, and clinical factors that influence PrEP uptake and retention in care among a sample of young, largely African American MSM enrolled in a PrEP program in the Deep South.

## Methods

### Sample and clinical setting

We interviewed 30 YMSM participating in a PrEP program at a clinic that provides preventive, primary care, and mental health services for Lesbian, Gay, Bisexual, and Transgender populations in Mississippi. The clinic also accepts PrEP referrals from a local sexually transmitted infections (STI) clinic that serves approximately 10,000 patients annually, including 3,000 sexual and gender minorities and 1275 AA MSM. PrEP care at the clinic is provided in accordance with Centers for Disease Control and Prevention (CDC) PrEP guidelines [34]. All participants in the current study were over 18 years of age, spoke and read English, and provided written informed consent. We interviewed 30 individuals retained in PrEP care as well as individuals who had not been retained in care. The University of Mississippi Medical Center’s Institutional Review Board approved this study.

### Data collection

Semi-structured interviews lasted approximately 45 minutes and assessed structural, social, behavioral, and clinical factors that may have affected individuals’ uptake, adherence, and retention in PrEP care. We used a purposeful sampling strategy; all participants were recruited after being prescribed and enrolling in the PrEP program. At the time of the interview, 25% of the sample had stopped taking PrEP or never filled their prescriptions. We phoned participants who had been lost to follow up care as many as five times to try to understand why they had discontinued PrEP. Interviews were conducted by trained qualitative researchers and conducted in a private room. Each participant received a $30 gift card. All interviews were digitally recorded and later transcribed. In accordance with grounded theory, we interviewed participants until we reached saturation, when no new data were being discovered.

### Data analysis

A general inductive approach guided the analysis of data, which allowed for the data to be formulated into themes and categories [35]. Coders read the transcribed data for participant responses that were similar and recurrent themes and patterns in the transcripts. Open and axial coding were then used to outline concepts among three coders. Each theme and sub-theme was assigned a code, and the codes were compiled in a codebook [36]. Quality checks were conducted on 20% of all transcripts via iterative coding by at least two coders. Discrepancies in interpretation were resolved among the research team before final coding commenced.

## Results

### Demographic characteristics

The mean age of participants was 26.6 years (SD = 8.2) and all participants were male. Most participants were AA (*n* = 25) and all reported being MSM. Most participants (*n* = 23) utilized the industry sponsored medication assistance program to help pay for PrEP medications; this program is only available to individuals who are uninsured and of low socioeconomic status. Seven of the 25 participants had not been retained in PrEP care and had discontinued taking PrEP. Among these individuals who had discontinued PrEP, more than half (n = 4) returned to PrEP care after their interview.

Across interviews, key themes emerged related to structural, social, behavioral and clinical factors that influenced PrEP use and retention in care (see Table 1). Structural factors affecting PrEP use included perceived limited access to payment assistance programs and concerns about costs of medications and clinical services. Social factors included HIV stigma and relationship status. Finally, behavioral factors included sexual risk behaviors and clinical factors included concerns about actual and perceived medication side effects. In addition, many individuals also reported positive spillover effects related to PrEP use; for example, several noted they felt more empowered to control their sexual decision-making and were more cognizant of their HIV acquisition risks than they were before starting PrEP.

**Table 1:** Primary results summarized.

Broad Factors Affecting PrEP Use	Themes and Quotes
*Structural Factors*	Access to payment assistance programs Copayments and deductibles for Medications and related services
*Social Factors*	HIV stigma and homophobia Relationship status changes
*Behavioral Factors*	Changes in sexual risk behaviors
*Clinical Factors*	Perceived and actual medication side effects
*Positive Spillover Effects*	Enhanced sexual health literacy More agency in sexual decision-making

### Structural factors

Many participants indicated that structural factors such as cost, assistance with medical visit and medication payments affected their experiences taking PrEP. While many participants noted that they had initially perceived the high cost of PrEP as a potential barrier to use, those barriers were overcome with the industry sponsored medication assistance program, which pays for PrEP for uninsured patients and provides assistance with medication copayments for insured individuals. One participant explained: *“It [the medication assistance program] covered most of my co-pay when I initially started PrEP*.*”* When asked how much participants would be willing to pay for PrEP, most participants reported they would be unable to pay for PrEP out of pocket, and would not be able to take PrEP if the medication cost were not subsidized. For instance, a participant stated: *“I wouldn’t pay more than fifty dollars*. *I probably would not be able to afford to pay out of pocket*.*”* Another participant reported that he had lost his job and health insurance, and because he did not know about the medication assistance program, he discontinued using PrEP:

“When I first started PrEP the doctor told me, I had to have insurance. At that time, I did have insurance, but when I switched jobs, I lost that insurance and it took a while to get it back. And when I went back to talk to her again, she said it was free now, so, but I never got up to talk to her again about restarting it.”

### Social factors

Social factors including participants’ relationship dynamics, their partners’ HIV serostatus and PrEP-related stigma also affected PrEP use and retention in care.

#### Relationship status and partner’s HIV status

Many participants stated that they believed they might not need PrEP while engaged in long-term monogamous relationships. A participant said: *“The only people that should be takin’ it are people who are not married*, *so when I get married*, *I shouldn’t have that problem*, *but up until that point*, *I will [take PrEP]*.*”* Other participants reported that they began using PrEP when they commenced a new relationship with someone who was HIV positive. One participant stated: *“I was in a relationship with someone that was positive*, *and I’m negative*. *We didn’t really use condoms all the time…and I got tested habitually but just so we could cover our bases*, *I decided to go on PrEP”*. Another participant noted how both medication cost and his relationship status affected his decision to continue taking PrEP:

“Who knows, maybe [I’ll be on PrEP] forever. Like I said, it depends on cost. I’m sure it’ll decline in cost over the years and there will be a generic out in probably ten years, There are things that will happen that will make it more affordable for more people in the future, but that’s hard to say. I may become monogamous. I doubt it, but…”

#### PrEP- related stigma

While most participants reported that they did not experience any external stigma related barriers to PrEP uptake, many stated that they worried about anticipated stigma from various sources. For instance, one participant was concerned about his parents noticing fees for PrEP-related medical services on his insurance statement. He stated:

“I was a bit worried about my insurance and my parents seeing it on my insurance bill, but then they had this little card that helps cover the cost that the insurance doesn’t pay for, and it doesn’t necessarily show up as PrEP that you’re taking on your insurance. So once I got over that, I was comfortable with taking it.”

Other participants relayed that they were concerned about their religious communities discovering they were taking PrEP. A participant who discontinued PrEP stated: *“Now I feel I just want to be honest to be myself*. *Why I didn’t take those pills*? *Because of my church*, *I think*.*”*

Additionally, some participants were concerned about how their sexual partners might react to them taking PrEP. For instance, one participant worried that his partner would question the trust in their relationship if he found out about his PrEP use: *“I was worried it might upset my partner that I was taking it because you should trust each other*.*”*

Another participant who discontinued PrEP noted that he had returned to attending religious services and decided not to have sex with men in the future, citing that he no longer needed PrEP.

### Behavioral factors

In addition to structural and social factors, many participants cited behavioral factors that influenced their PrEP use, such as sexual risk behaviors. Some mentioned they met anonymous partners online or had multiple sexual partners, and that these actions resulted in their consideration of PrEP use. One participant stated: “*I did [meet partners online]*. *That is the first time I did*. *That’s what I did and it really hurt me bad…*.*I met someone online*, *and we performed unsafe sex*. *And I worry about my status every day*. *Every single day*. *I never stop worrying about that*.*”* Other participants stated that they started taking PrEP because they were having sex with multiple people. For instance, one participant reported: “*I got on PrEP because I thought it was really beneficial for me because having sex with multiple sex partners was putting me at risk*.*”*

### Clinical factors

Most participants reported no side effects or clinical challenges associated with PrEP use; only two participants experienced or perceived side effects that prompted them to discontinue PrEP. For instance, one participant reported stopping PrEP after experiencing nightmares:

“I was having nightmares. I wasn’t experiencing these until I started PrEP, but I think it was because it was like stress plus the side effects, which made it worse. I didn’t like it. It was fine taking a pill every day. I always stayed on task. I took it at the same time every day, but [because of the nightmares] it wasn’t fitting.”

Another participant noted: *“The only side effect that I experienced was a bad headache when I first started takin’ it*, *and being nauseous*. *It lasted for the whole day*. *But After I took it [PrEP] the first time I stopped*.*”*

Still, *anticipated* side effects were a major concern for many participants, and for some, were a factor undermining retention in PrEP care. One participant said: *“As long as we continue to determine that there are no side effects*, *I’ll continue*. *But I worry about my liver*, *I worry about my kidneys*.*”*

### Positive spillover effects of PrEP uptake

Many participants noted the positive impacts taking PrEP had on their overall and sexual health. Many reported that taking PrEP had prompted them to be more discerning when choosing their sexual partners. One participant stated: *“I’m more cautious [about picking my partners] now*.*”* In addition, some participants reported discussing risk reduction practices more with their sexual partners after they commenced PrEP. One participant reported: *“I feel safer when doing it [having sex]*, *and it has made me more aware when it comes to my sexual habits*. *I ask more questions now*.*”* Most participants reported that they did not change their risk behaviors after commencing PrEP; most reported either no change or an increase in condom use after starting PrEP. A participant stated: *“I really feel like I’ve won my life back*. *I don’t want to put my life at risk anytime again*, *and the doctor told me I can take this pill*, *but I still need to have safe sex*.*”* One participant also noted that taking PrEP allowed him to continue to enjoy sex: *“As long as I take these pills*, *I can still enjoy sex*.*”*

### Opportunities for re-engaging patients in PrEP care

Finally, interviewing individuals who had fallen out of PrEP care prompted four participants to re-engage in PrEP care. One participant previously unaware of the medication assistance program enrolled in the program after his interview. Another participant in a serodiscordant relationship who had discontinued PrEP also underwent HIV testing the same day as the interview, and ultimately re-engaged in PrEP care. These success stories suggest that individuals who are lost to PrEP care can often be re-engaged in PrEP care.

## Discussion

To our knowledge, this is among the first studies to explore the lived experiences of YMSM prescribed and taking PrEP in the Deep South. Our findings suggest that structural factors such as insurance, costs and copayments as well as social factors such as relationship dynamics and stigma impacted PrEP uptake and retention in PrEP care. Behavioral factors including sexual risk behaviors and clinical factors such as actual and perceived side effects also affected participant’s decisions about starting and continuing to take PrEP. Additionally, participants reported many unintentional positive, health-related spillover effects of taking PrEP and many of the MSM who had discontinued PrEP use re-initiated after participating in this study.

Our results echo findings from other studies related to the barriers and facilitators of PrEP programs [37–40]. A recent qualitative study including 24 predominately AA MSM in Los Angeles also demonstrated that stigma affected PrEP initiation and adherence [37]. Recent research by Philbin and colleagues also highlighted stigma as a possible multi-level factor that can affect attitudes and uptake of PrEP among AA MSM [31]. Participants in the this study were recruited from New York City, and the social stigma associated with identifying as MSM in the Deep South may be even greater.

Previous research has also found that *perceived cost* may be a barrier to PrEP uptake. For instance, a 2013 study reported that many YMSM believed they would not be able to afford PrEP if they lacked insurance coverage [24]. Although, another recent study found that *actual* cost of PrEP was not a factor that affected PrEP retention in care [41]. Perceived and actual costs of both PrEP medications and clinical visits undermined retention in PrEP care in the current study. Although most participants utilized the medication assistance program that helped pay for PrEP care, many worried about being able to continue on PrEP if the financial assistance not available. Finally, individual sexual risk behaviors also influenced PrEP uptake and adherence in this population, as in other studies [40].

Findings from this study suggest that structural, social, behavioral and clinical factors should be considered when designing and evaluating PrEP programs focused on engaging AA YMSM in the Deep South. Additionally, patient education that includes detailed information about patient assistance programs and the possible side effects of PrEP would likely enhance PrEP uptake. This strategy aligns with previous literature that highlights the need for public policy and social marketing sources to address stigma associated with PrEP use in order to promote PrEP uptake and retention in PrEP care [42, 43]. In the Deep South, stigma related to sexual orientation and sexual behavior may pose challenges to PrEP uptake and retention in PrEP care; social marketing, public policy and PrEP programs should respond to these contextual challenges.

It is also noteworthy that over half of the individuals who had discontinued PrEP care indicated that they were interested in taking PrEP again after their interview. This demonstrates the importance of consistent outreach for individuals enrolled in PrEP programs, particularly those who are not retained in care, as well as the opportunity to re-engage patients with case management services. Intensive case management that assists patients with overcoming barriers to care has proven effective for individuals living with HIV and at-risk groups [44–47].

Finally, novel in our findings is that many participants reported being more attuned to their health and well being after starting PrEP. Participants stated that they were “more cautious”, “more aware”, and had won “their life back” and many engaged in less condomless sex while on PrEP. This positive spillover effect of PrEP use has implications for practice. For instance, those who successfully initiate PrEP could act as PrEP peers to educate other at-risk groups about the cascading effect of PrEP use on general well being. The dissemination of this knowledge could help eliminate known PrEP uptake barriers and social stigma specifically linked with PrEP use.

### Limitations

This study is subject to several limitations. While we interviewed some patients who were not retained in PrEP care, several others were completely lost to follow up and were not included in our sample. Our findings may therefore not represent the experiences of all patients who were not retained in care, or the broader US. Our study does present important findings identifying barriers to uptake and retention in PrEP care for individuals who are among the highest risk for HIV acquisition in the US.

## Conclusion

Addressing structural factors such as cost or access to payment assistance programs, social factors such as stigma and relationship dynamics, and clinical and behavioral factors such as anticipated or experienced side effects of PrEP medication and sexual risk behaviors is imperative. With persistent outreach, we were able to re-engage several patients in PrEP care who had previously been lost to follow-up, suggesting that barriers to retention in PrEP care can be overcome in the Deep South. Future research to promote PrEP uptake and retention in care should address social, structural, behavioral and clinical factors.
